# Challenges to Successful Implementation of the Immune Checkpoint Inhibitors for Treatment of Glioblastoma

**DOI:** 10.3390/ijms21082759

**Published:** 2020-04-16

**Authors:** Stephanie Sanders, Waldemar Debinski

**Affiliations:** 1 Department of Cancer Biology, Wake Forest School of Medicine, Winston Salem, NC 27157, USA; svsander@wakehealth.edu; 2 Brain Tumor Center of Excellence, Wake Forest Baptist Medical Center Comprehensive Cancer Center, Winston Salem, NC 27157, USA

**Keywords:** glioblastoma, immune checkpoint inhibitors, immunotherapy

## Abstract

Glioblastoma (GBM) is the most common and aggressive malignant glioma, treatment of which has not improved significantly in many years. This is due to the unique challenges that GBM tumors present when designing and implementing therapies. Recently, immunotherapy in the form of immune checkpoint inhibition (ICI) has revolutionized the treatment of various malignancies. The application of immune checkpoint inhibition in GBM treatment has shown promising preclinical results. Unfortunately, this has met with little to no success in the clinic thus far. In this review, we will discuss the challenges presented by GBM tumors that likely limit the effect of ICI and discuss the approaches being tested to overcome these challenges.

## 1. Introduction

Glioblastoma (GBM) is the most common and aggressive malignant glioma and is characterized by uncontrolled cellular proliferation, increased necrosis, diffuse brain infiltration, and high vascularity [1,2]. Most GBM tumors develop *de novo* and are classified as primary tumors, but a small subset arises from a progressing low-grade glioma, which is termed a secondary GBM [3]. Secondary GBM tumors account for 5% of GBM diagnoses and all of them have isocitrate dehydrogenase (IDH) mutations [4,5]. Patients diagnosed with GBM have a median survival of 15–17 months and patients with secondary GBM have the best prognosis [3]. Treatment of GBM has remained relatively unchanged for many years and consists of surgical resection of the tumor followed by radiation and administration of temozolomide (TMZ). GBM tumors are sensitive to TMZ treatment in direct correlation with the levels of methyl guanine methyl transferase (MGMT); however, tumors recur inevitably in practically all cases [6] and higher doses of the chemotherapeutic did not show any clinical effect at all [7]. Recurrent tumors can also be treated with bevacizumab (Avastin), which targets VEGF-A [8,9], or with recently approved tumor treating fields (TTFs) that are emitted by wearable devices in the form of electric impulses disrupting tumor cell division [10]. Treating GBM is much more challenging than other solid tumors because of the blood–brain barrier (BBB) which will be discussed in further detail below. Briefly, the BBB isolates the tumor from therapeutic accession by creating a selectively permeable barrier around most central nervous system (CNS) blood vessels [11,12]. GBM also has a so called blood–brain tumor barrier (BBTB) due to abnormal neovasculature with irregular blood flow further preventing drugs from exiting the circulation which has an effect on the treatment of the tumor when drugs are delivered systemically [11].

Modulation of the patient’s immune system has become an increasingly utilized method to combat various types of cancer [13]. Unfortunately, immune modulation has shown limited success in GBM thus far. However, immune modulation has shown some initial promise as an adjunct therapy for GBM [14,15,16]. Various immune treatments such as dentritic cell or tumor antigen vaccines or immune checkpoint inhibition have been tested in the clinic [17]. Immune checkpoint inhibition has become widely used as it has been successful in a variety of different cancers [13,18,19,20,21]; therefore, it is important to understand how immune checkpoints work and how the different FDA-approved ICIs work to block those checkpoints.

There are two general pathways by which effector T cells are stimulated to enact their cytotoxic function. The first involves presentation of an antigen associated with an MHC to the T cell receptor (TCR), while the second involves costimulatory signaling [22]. One of the most prominent costimulatory signals occurs when CD80/86 present on antigen presenting cells (APCs), some macrophages, dendritic cells, and other activated leukocytes binds to the CD28 receptor on the surface of T cells [23]. This stimulatory signal includes increased interleukin 2 (IL-2) production and IL-2 receptor expression, which results in increased proliferation and differentiation into cytotoxic T cells [24]. Cytolytic T-lymphocyte associated protein 4 (CTLA4), a protein expressed on the surface of activated T cells [25], acts as a checkpoint to regulate T cell responses upon binding of its ligands [26]. It shares a 75% nucleotide sequence homology with the stimulatory receptor CD28 and in fact binds the same ligands, CD80 and CD86; however, CTLA4 binds with higher affinity to CD80/86 than CD28 does [23]. In fact, CTLA4 binds to CD80 or CD86 with a Kd of 12 nM [27] while CD28 binds the ligands with a Kd of 200 nM [28]. Binding of ligands to the CTLA4 receptor has several effects. First, it acts to sequester CD80/86 away from CD28, thereby preventing stimulatory signaling. Evidence suggests that the binding of ligands to CTLA4 may block cell cycle progression, preventing the proliferation of T cells [23]. Second, CTLA4 has also been linked to promoting regulatory T cell function [29] and has been found to be a key negative regulator of T cells. In fact, mice born without CTLA4 suffer from lymphoproliferation and die at only four weeks of age [18].

Targeting CTLA4 in order to release the suppression of the immune system has demonstrated efficacy in the oncology clinic [19,20], meaning that this mechanism of immune system inhibition is widely operational among malignancies. Ipilimumab is an FDA-approved monoclonal antibody that targets the CTLA4 receptor and blocks its interaction with CD80/86, thereby releasing the suppressive signaling and allowing cytotoxic T cells to perform their effector function [21]. In fact, treatment of patients with metastatic melanoma with Ipilimumab showed an increase in overall survival of four months when compared to control treatment [21]. CTLA4 blockade also showed efficacy when combined with immune-stimulatory treatments such as gene viral or radio-therapy [18]. Unfortunately, CTLA4 blockade alone will be insufficient to promote an enhanced immune response as it is only present on the surface of T cells after their stimulation [30] and GBM is known to be an immunologically suppressed tumor; therefore, blockade of an additional immunosuppressive receptor(s) not dependent on T cell activation should result in even more effective anti-tumor action.

Programed Death 1 (PD1) is a surface receptor expressed on T cells which has been associated with poor prognosis when expressed in human tumors [30]. There are two ligands for PD1 known as PD-L1 and PD-L2 [30]; binding of these ligands to the PD1 receptor results in immune suppression. This immune suppression occurs via apoptosis or exhaustion of T cells while blocking of this interaction enhances anti-tumor immune activity [16]. PD1 null mice showed autoimmune disorders that are organ specific and strain dependent. However, unlike CTLA4 null mice, PD1 null mice do not develop these disorders until after six months of age and the autoimmune disorders are restricted to certain organs rather than being systemic [30].

The ligands for PD1 are commonly expressed in many tumors including on the surface of GBM tumor cells [31]. Therefore, targeting the PD1 receptor on T cells may help to prevent the tumor-mediated immune suppression. Multiple clinical trials have demonstrated the efficacy of targeting PD1 as a therapy for various types of tumors including lung, bladder, breast, and pancreatic cancer to name a few [31,32]. Furthermore, when patients with advanced melanoma, non-small cell lung cancer (NSCLC), or renal cell cancer were treated with PD1 monoclonal antibodies, Phase III clinical trials showed increased efficacy of treatment, as well as improved patient quality of life [33,34,35,36]. This has started a highly successful application of ICIs in a variety of cancers, which in a way has recently revolutionized cancer treatment [13,37]. The current FDA-approved ICIs are summarized in Table 1. In this review, we will discuss the barriers GBM presents for using ICIs, mechanisms for bypassing these barriers, the current preclinical work being performed, as well as current clinical trials involving the use of ICIs in GBM treatment.

## 2. Barriers to ICI Use in GBM

GBM presents several challenges when designing and implementing therapeutics; these challenges include the presence of the BBB and BBTB, heterogeneity of tumors, the immunosuppressive microenvironment of GBM, as well as its highly infiltrative nature. When attempting to treat GBM with immunotherapeutics, it is important to consider each of these challenges and implementing methods to compensate for them.

### 2.1. Blood–Brain Barrier

The concept of a selective barrier between the blood and the brain was first demonstrated in 1885 when a study involving the infusion of Evan’s blue dye intravenously into a rat showed that all organs stained except the brain [38]. However, it was not until 1913 when a study in which dye injected into the cerebral spinal fluid (CSF) showed that only the brain tissue stained, that it was made clear that a barrier exists between the blood and the brain [38]. In fact, there are three “barriers” that exist to protect the brain from unwanted natural internal compounds, foreign substances, as well as to maintain homeostasis. These include the blood–brain barrier, the blood–CSF barrier, and the CSF–blood barrier [38,39]. When treating tumors like GBM, the most important barrier to consider is the BBB and a pathological BBTB.

The BBB protects the brain from pathogens and controls the immune regulation and infiltration into the brain from the circulatory system [38]. It is comprised primarily of the endothelial cells of the blood vessels within the brain itself. These cells have an increased number of tight junctions which tightly adhere the cells together and do not allow ions, proteins and many circulating cells to enter the brain [38]. These endothelial cells are covered by astrocyte foot processes that provide communication between the brain and the circulatory system. Astrocytes can release signaling molecules which will modify the permeability of the BBB in order to regulate the entrance of a cell or compound into the brain [39]. This allows for control of water homeostasis, prevents tissue damage and controls electrolyte flux within the brain [38].

The endothelial cells of the BBB have transporters which allow for nutrients to exit the blood and for waste products in the brain to be removed [38]. For example, GLUT1 is a glucose transporter which is expressed at the BBB and allows for glucose and other hexose sugars to enter the brain and be used as an energy source [40]. The BBB also contains ion transporters to allow for transport of ions such as sodium, potassium and calcium both into and out of the brain [39]. There are also transporters and receptors which mediate the passage of signaling molecules like cytokines and chemokines both into and out of the brain in a process termed transcytosis [40].

While the BBB does encompass the majority of the brain, there are a few areas in which it is absent, namely the circumventricular organs such as the pineal gland [38]. These areas of the brain are unique in that they require cross-talk between the brain and peripheral blood as they are involved in releasing and transporting hormones and other systemic signaling molecules [38]. It is important to note that while the BBB is not present in these areas of the brain, substances that enter the brain in those locations are prevented from spreading to protected areas by other specialized epithelial cells [38].

The presence of a BBB, which tightly regulates the entry of therapeutics into the brain and brain tumor environment, presents a challenge to treatment of brain malignancies in that it becomes more difficult to control the diffusion of a therapeutic into the brain tumor itself. Therefore, methods that alter BBB permeability or bypass the BBB altogether are important to consider when utilizing therapeutics to treat such malignancies. This is especially important for the use of ICI therapy as its efficacy depends not only on the blocking of immune checkpoints but also on the subsequent infiltration of immune effector cells into the brain tumor and their targeting there.

#### Bypassing the BBB

There are a few ways to bypass the BBB when designing a treatment targeting GBM. The first is to design a therapeutic to be more amenable to passing through the endothelial cells that make up the BBB. This can be accomplished by making an agent more lipophilic, lower its polarity or reduce its ability to form hydrogen bonds [39]. In general, nonpolar, lipophilic compounds that are <400 Da are able to passively diffuse across the membranes of the endothelial cells in the BBB [39]. Compounds that do not fall into this category must utilize active transport across the BBB if delivered systemically. It is important to note that the BBB also contains drug efflux pumps such as P-glycoprotein (ABCB1), which can act to block lipid-soluble drugs from entering the brain [39].

A second method of bypassing the BBB is using the “Trojan horse” method [39]. In this method, a compound which normally would be unable to pass into the brain is conjugated to a monoclonal antibody against one of the transcytosis receptors on the BBB. The binding of the monoclonal antibody to the receptor will trigger the endothelial cell to grant entry into the brain, thereby allowing the compound to sneak in undetected [40]. Additionally, utilizing nanotechnology to deliver therapeutics has shown some success as a method for crossing the BBB. For example, using a liposome with an antibody targeting transferrin as a carrier for a therapeutic agent allows for the agent to be carried across the BBB and infiltrate the tumor [41]. Inorganic nanoparticles (IONPs), which have an iron oxide core, can both deliver a therapeutic agent to the brain while also acting as an imaging agent for MRI; this allows for tracking of the delivery of therapeutic agents to the tumor itself [41]. A third method utilizes targeted peptides to pass through the BBB and home to the tumor; these peptides can be conjugated with a therapeutic molecule in order to deliver treatment directly to the tumor without harming the surrounding brain [42]. For example, Pep-1L is a peptide which is targeted to interleukin 13 receptor alpha 2 (IL13RA2), a receptor found on the surface of GBM cells and not in a normal brain [43,44]. This peptide was found to accumulate in GBM intracranial xenograft tumors in mice [43]. In a murine study utilizing CED of Actinium-225 conjugated Pep-1L, the conjugate was found to be well tolerated and increased overall, median and mean survival compared to control mice [45]. A separate study showed that conjugating Pep-1 to the surface of a nanoparticle (Pep-NP) allowed the nanoparticle to infiltrate GBM tumors when administered systemically in mice [46]. These studies show that targeted peptides can be used to deliver therapeutic agents directly to the GBM tumor without affecting the surrounding brain tissue.

Physical disruption of the BBB will also allow drugs to enter the brain; this can be accomplished with radiation, electroporation, low intensity ultrasound, among others [47,48,49,50]. A Phase I/IIa clinical trial was recently completed in which low-intensity pulsed ultrasound (LIPU) was used to disrupt the BBB and allow entry of a drug into the brain. The trial involved the implantation of the SonoCloud-1 device into the skull bone of patients through which pulsed sonication could be delivered. The study showed that LIPU was well tolerated in patients and that, following sonication, carboplatin was able to enter the brain [48]. Irreversible electroporation (IRE) has been shown to both disrupt the BBB as well as ablate tumor cells in a canine model [36]. This technique has been modified to overcome the limitations discovered in testing of the IRE system; the modified technique is called high-frequency irreversible electroporation (H-FIRE). In this system, electrodes are placed into the tumor in order to deliver short bursts of bipolar pulses. These pulses increase the transmembrane potential in cells, creating pores in the membranes and leading to cell death [51]. The H-FIRE system has been tested in canines who developed spontaneous meningiomas and was well tolerated with no reported damage to the surrounding brain [51]. Finally, the use of stereotactic radiation has been part of the standard of care for GBM treatment for many years; it has the added effect of increasing the permeability of the BBB as endothelial cells are also susceptible to ionizing radiation [52]. Each of these methods would allow drugs delivered systemically to pass through the BBB and enter the brain.

Finally, delivering a therapeutic intracranially allows for circumvention of the BBB as intratumoral delivery does not rely on circulation for drug delivery and therefore avoids the BBB altogether. To that end, there are already some methods used in the clinic which employ this strategy. The first is the use of Gliadel^®^ wafers to deliver BCNU directly onto GBM tumors. The Gliadel^®^ wafer is approved for the treatment of both primary and recurrent GBM and is usually implanted into the resection cavity at the time of surgery [47,50]. Currently, a new polymer wafer system is in development which combines BCNU with TMZ treatment in one wafer in order to deliver combinatorial therapy to GBM tumors [50].

Additionally, convection enhanced delivery (CED) bypasses the BBB to deliver drugs directly to the tumor or in the vicinity thereof. First described in 1994, CED is a catheter based system for intratumoral delivery of therapeutic compounds to the brain [53]. This method involves the use of strategically placed catheters to deliver treatment intratumorally; CED allows clinicians to target the tumor as a whole, as well as the surrounding parenchyma, in order to treat areas of tumor invasion which may be difficult to resect surgically [54]. A comprehensive review of CED discussed the details of various catheter systems used and how CED can be used in the clinic to deliver treatment to GBM tumors [55]. CED has several advantages over other approaches including bypassing the BBB, directed drug delivery to tumors, and the ability to target disease that is located deep within the brain and difficult or impossible to excise. Being that CED is based on direct drug delivery to tumors, many systemic side effects can be avoided [56]. The use of CED to deliver treatment to GBM tumors has shown encouraging results in the clinic as well. A targeted, truncated form of Pseudomonas exotoxin, A (PE), was infused via CED in an efficacy trial and was found to be safe but the clinical results have not been matched since [55,57,58].

By designing treatments that are more amenable to passing through the BBB, disrupting the BBB or bypassing the BBB altogether, ICIs will be better able to reach the immune checkpoint receptors that are expressed in the GBM tumor microenvironment, along with allowing more effector cells to reach the tumor itself. The use of CED would allow more control of the delivery and concentration of ICI, which interacts with the tumor and may improve treatment outcomes in patients with GBM.

### 2.2. Immune Infiltrate in GBM Influences Immune Evasion

Infiltration of immune cells in the GBM microenvironment is important for the use of immunotherapies, especially immune checkpoint inhibition. It is widely agreed that GBM tumors possess a highly immunosuppressive microenvironment which is maintained not only by tumor cells themselves but also by the immune infiltrate [59,60,61]. There are a variety of immune cell types present in GBM tumors and they each contribute to either promotion and progression or tumor inhibition. This contribution to immune suppression includes expression of immune checkpoint receptors like CTLA4 and PD1; by understanding how and where immune cells infiltrate the GBM microenvironment, we can better design treatment strategies which utilize ICIs to modulate the immune response to GBM tumors.

#### 2.2.1. Lymphocyte Infiltration

Tumor infiltrating lymphocytes (TILs) vary widely in GBM with some tumors having high infiltration while most exhibit low numbers [59]. There is evidence to suggest that low infiltration of T cells into GBM tumors may be due to sequestration of T cells in the bone marrow [62]. Both CD4^+^ and CD8^+^ T cells are present in GBM tumors and, frequently, these cells are also expressing immune checkpoint receptor PD1 [60]. There is evidence suggesting that the T cells present in GBM are “defective” in that they have reduced proliferative activity and reduced ability to produce IL-2 [63,64]. Patients with GBM also present to the clinic with lymphopenia and have significantly reduced CD4^+^ T cell counts; however, the fraction of CD4^+^ cells which are regulatory T cells (T regs) is increased in GBM patients [63]. These T reg cells contribute to the defective proliferation of CD4^+^ T cells and depletion of T reg cells in a murine model led to rejection of the tumor [63]. This suggests that T reg cells are important mediators of immune response to GBM and are likely contributing to immune evasion. Recruitment of T reg cells has been correlated with IDO1 expression in GBM tumors as well as poor overall survival in GBM patients [65]. IDO1 is involved in tryptophan catabolism; it is thought that inaccessibility of tryptophan leads to inhibition of T cells [66].

#### 2.2.2. Macrophage and Microglial Infiltration

Macrophages and microglia can be hard to differentiate as no unequivocal marker exists to distinguish them. However, it is suggested that the presence of CD49D is indicative of a bone marrow-derived macrophage (BMDM) while the absence of CD49D indicates a brain resident microglial cell [60,67]. Using this marker, it has been shown that macrophages and microglia have different patterns of infiltration into the GBM microenvironment [60]. Macrophages appear to be more prevalent in the core of the GBM tumor and less present in the margins while microglia have the inverse pattern, being less present in the core of the tumor and more present in the margins [60]. Recent reports have identified new markers for microglial cells including TMEM119. These results have been confirmed via RT-PCR and immunohistochemistry [68]. These new markers may help to further evaluate differences in the roles of macrophages and microglia within GBM tumors [68]. Gene expression patterns in macrophages depend heavily on the microenvironment in which the macrophages are present [69]. GBM associated macrophages (GAMs) express genes related to wound healing, immune suppression, as well as MHC-II and costimulatory signaling genes [67]. This suggests that GAMs are present in a chronic wound healing state generally attributed to the alternatively activated (M2) macrophage [67]. The macrophages and microglia that infiltrate GBM tumors have been shown to contribute to the immune suppressive microenvironment within these tumors [60,63,67,69].

### 2.3. Immune Suppressive Microenvironment in GBM

As mentioned previously, GBM has an immunosuppressive microenvironment. This is partially explained by the cytokines and secreted signaling molecules produced by T regs, macrophages, and microglia within the tumor microenvironment [59,60,63,67,69]. However, another contributing factor is the presence of immune checkpoint signaling within GBM tumors. GAMs have increased expression of PD-L1 compared to circulating monocytes [60]. PD-L1 expression within GBM tumors is thought to be due to PTEN loss as PD-L1 expression is regulated by Akt and loss of PTEN leads to constitutive activation of Akt [61,70]. These results need to be confirmed in GAMs to elucidate the mechanism behind increased PD-L1 expression. Increased levels of PD-L1 found in GBM tumors lead to an increase in apoptosis of T cells [60]. GBM cells, as well as conditioned media, have been shown to be sufficient to promote this increase in PD-L1 expression on the surface of monocytic cells [60]. PD1 expression is most commonly found on the surface of T cells, especially activated T cells [71]. Evidence suggests that it is the interaction between GAMs and TILs which primarily mediates the PD1/PD-L1 axis of immune suppression [72]. Additionally, CTLA4 expressed on activated TILs also acts as a mechanism for immune evasion in GBM [63].

The expression of several immune checkpoint proteins including CTLA4, PD1 and PD-L1 within the GBM microenvironment provides a context in which ICI therapy would be an effective treatment. However, it is important to consider all of the barriers mentioned here when implementing such therapy if we hope to achieve a successful outcome.

## 3. Clinical Experience with Using ICIs to Treat GBM

Despite encouraging preclinical data, response to immune checkpoint blockade in the clinic has been disappointing when treating GBM. In the recent Checkmate 143 trial (NCT02017717), combinatorial Ipilimumab/Nivolumab treatment was compared with Nivolumab alone in patients with recurrent GBM [73]. The Phase I trial found that Nivolumab alone was better tolerated in patients than combinatorial therapy and this led to a Phase III trial in which an FDA-approved bevacizumab treatment with or without nivolumab was investigated in patients with recurrent GBM. Unfortunately, this trial failed to meet its primary endpoint, the overall survival (OS), as patients treated with Nivolumab did not live longer than those treated with bevacizumab. In fact, the objective response rate to Nivolumab treatment was lower than that to bevacizumab treatment, though patients who responded to Nivolumab treatment had a median duration of response at 11 months compared to bevacizumab at five months [74].

There are a few potential reasons for the failure of the CheckMate 143 trial. The BBB may still pose an issue to systemic delivery of ICIs considering that T cells in the periphery are the primary target of systemically delivered ICIs [14]. Many T cells in the peripheral blood may remain unactivated and may never enter the brain or the brain tumor. With systemic treatment, ICIs may bind to peripheral T cells that never enter the brain or the tumor microenvironment and cannot reach tumor-residing T cells. In addition, simply targeting one immune checkpoint, as was the case with CheckMate 143, may not be sufficient. GBM is known to be highly immunosuppressive and may still be able to suppress cytotoxic T cells by different checkpoints. Or, even when activated, the T cells cannot effectively penetrate tumor microenvironment and/or maintain the anti-tumor activity while in tumor.

A retrospective evaluation of the use of Pembrolizumab in the treatment of recurrent CNS tumors, including GBM, was not encouraging either. The study demonstrated that patients treated with Pembrolizumab did not experience improved survival with treatment [75]. In view of the failure of ICIs in the clinic, a Phase II window-of-opportunity trial (NCT02337686) was conducted to assess the immune effector cells upon treatment with an ICI [76]. This study examined the relative numbers of CD3^+^ lymphocytes and CD68^+^ monocytes in tumors in order to determine the composition of the immune infiltrate in recurrent GBM tumors after treatment with Pembrolizumab. It was found that there were more CD68^+^ cells present than CD3^+^ cells with CD68^+^ cells making up 72% of the immune cells and CD3^+^ cells making up 22% of immune cells in the Pembrolizumab-treated GBM tumors tested. This is indicative that there are more macrophages in recurrent GBMs rather than T cells tipping the balance towards immunosuppression in GBM, even with ICI treatment. The authors thus hypothesized that the high presence of macrophages may be interfering with efficacy of ICI therapy [76]. This trial highlights the importance of understanding the immune infiltrate of both primary and recurrent GBM when considering how and when treatment is administered.

There is a currently ongoing clinical trial, which endeavors to overcome the BBB by delivering ICIs directly into the resection cavity of recurrent GBM tumors. The GlitIpNi trial (NCT03233152) tests Ipilimumab monotherapy or Ipilimumab in combination with Nivolumab injected directly into the resection cavity at time of surgical resection of the tumor. Both cohorts also receive intravenous (IV) Nivolumab starting pre-operatively for a maximum of six doses. Treatment has been generally well tolerated with only two patients experiencing grade 3 increase in cerebral edema with neurodegenerative symptoms which were reversible upon steroid treatment [67]. One patient developed an inflammatory intracerebral cyst. Median follow-up indicated PFS at 14.4 weeks with one-year overall survival (OS) of 46% and two-year OS at 15% [77]. A separate study used electronic records from 50 patients with GBM to assess OS and PFS in an effort to estimate national OS and PFS using routinely collected data. This study reported that patients treated with chemotherapy and radiotherapy had a median PFS of 7.4 months and median OS at 12.8 months or 68% one-year OS [78]. Therefore, the numbers reported in the GlitIpNi trial are not encouraging. It is likely that a passive diffusion of the ICIs from the resection cavity into the surrounding brain is limited hence a relative lack of effect using this approach is not unexpected.

The currently active clinical trials for GBM using ICIs are summarized in Table 2. Most of the trials involve combinatorial therapy in which the ICIs are combined with other immunostimulatory therapies [79]. For example, a Phase I trial (NCT03422094) is investigating the safety and feasibility of stimulating a neoantigen-specific T cell response paired with Nivolumab treatment [80]. Similarly, a separate Phase I trial (NCT03576612) explores the use of gene mediated cytotoxic immunotherapy (GMCI) to stimulate an immune response and combines it with an ICI to block suppression of that immune response [81]. GMCI takes the form of aglatimagene besadeovec (AdV-tk) injection into the tumor cavity at time of surgical resection, followed by oral valacyclovir three times a day for two weeks to kill tumor cells to produce an in-situ vaccination effect [81]. Patients also receive standard of care therapy in the form of radiation and temozolomide, as well as IV Nivolumab every two weeks until 52 weeks, disease progression, or unacceptable toxicity [81]. In both of these studies, the investigators are taking the immune infiltrate as well as the immunosuppressive microenvironment into account. In one study, the neoantigens will be delivered systemically and are expected to produce a targeted immune response to the tumor which will be enhanced by blockade of the PD1 immune checkpoint receptor. In the GMCI study, the BBB is bypassed at time of surgical resection to deliver an immunostimulatory therapeutic whose immune response will again be enhanced by the blockade of immune checkpoints.

There are two trials that explore the addition of ICIs to standard of care therapy only. One such trial is assessing a PD-L1 inhibitor in newly diagnosed GBM (NCT03047473) [82]. In this Phase II study, Avelumab, a PD-L1 inhibitor, is administered via IV injection within three weeks of finishing radiotherapy [82]. Secondary endpoints of the study include using iRANO criteria to grade tumor response and evaluating potential biomarkers of response [82]. Additionally, a Phase II clinical trial (NCT027984) in recurrent GBM explores the use of a genetically modified oncolytic adenovirus DNX-2401 injected directly into the tumor via cannula followed by IV administration of Pembrolizumab; Pembrolizumab is given every three weeks for up to two years or until time of disease progression [83]. The study measures objective response rate with secondary outcomes of overall survival, time to tumor response, and duration of response up to 3.5 years [83]. While the Avelumab study does not specifically address any of the barriers presented in this review, it will provide data on the use of a new ICI in newly diagnosed GBM and has the potential to reveal biomarkers which will aid in tracking treatment efficacy. The DNX-2401 study addresses the BBB by utilizing intratumoral delivery of the virus to stimulate an anti-tumor immune response while attempting to sustain that response by blocking the PD-L1 immune checkpoint.

Another currently active Phase I/II clinical trial (NCT03277638) examines tolerability and efficacy of using Laser Interstitial Thermotherapy (LITT) in combination with Pembrolizumab to treat recurrent GBM [84]. There are three arms in this study with the major difference between arms being the time to treatment with Pembrolizumab. All patients will receive LITT which is a surgical procedure that uses a laser beam to heat tumors and ablate tumor cells. This technique will bypass the BBB and is also expected to induce immune response which will be sustained with ICI treatment. In the first arm, patients receive IV Pembrolizumab seven days prior to surgery, in the second arm, Pembrolizumab injections begin 14 days after surgery, while in the third arm Pembrolizumab injections begin 35 days after surgery [84]. The primary outcomes for this trial are the optimal timing and safety of LITT with Pembrolizumab and tumor response to the therapy. Secondary outcomes include progression free survival, and overall survival [84].

Finally, a Phase II clinical trial (NCT03890952) is assessing PD-L1 and other immune biomarkers which may be predictors of anti-tumor activity of Nivolumab in recurrent GBM [85]. The two arms in this study examine combinatorial Nivolumab and Bevacizumab in patients not undergoing salvage surgery versus patients undergoing salvage surgery. In this study, the primary outcome is the number of insertions and deletions in tumor versus germline control samples. The investigators will map cancer-specific mutations, and splice variations and predict the T cell epitopes overlapping these regions [85]. In doing so, the investigators hope to determine important immune-related biomarkers which may predict response to Nivolumab treatment in patients with recurrent GBM. The secondary outcome for this study is PFS in both treatment arms [85].

Though past clinical trials utilizing ICIs to treat GBM have shown little to no efficacy, there is still hope for changing it. Clinical trials in patients with metastatic melanoma with ICIs showed their presence in the CSF, along with responses of metastatic brain lesions, indicating that the ICIs had crossed the BBB and can produce effects in brain malignancies [86,87]. This is a very encouraging development that needs to be reproduced in other brain metastases.

The failure of recent trials suggests that the barriers discussed earlier are impacting the efficacy of ICIs in GBM indeed. Current clinical trials will help to expand knowledge of how ICIs can be better incorporated as a treatment for GBM and many of them are designed such that they circumvent at least one barrier that GBM tumors present. The window of opportunity trial provided valuable insight into the immunosuppressive microenvironment present in GBM tumors during treatment with an ICI [76]. This is important in order to discern how best to abrogate the immune suppression present in the GBM tumor microenvironment. Two of the trials discussed here will use immune stimulatory therapy in combination with an ICI [80,81] which may help to overcome the result of the window of opportunity trial by preventing activated, tumor-targeted T cells from being suppressed by the tumor. Additionally, some of the trials discussed above utilize loco-regional delivery of the therapeutic, which offers bypassing the BBB/BBTB completely and allow for more control of the concentration of therapeutic delivered directly to the tumor [83] or the resection cavity [77,81,84]. These clinical trials provide hope for more effective use of immunotherapeutics in the treatment of GBM as new strategies are also investigated in a preclinical setting.

## 4. Experimental Attempts at Making ICIs Work in GBM

Combinatorial immunotherapy has become a major part of clinical investigation in the context of immunotherapy for treatment of GBM, as discussed above. This therapeutic strategy is also under investigation in a preclinical setting like combinations of ICIs along different immunotherapy approaches, such as dendritic cell vaccination, in order to sustain or bolster the immune mediated killing of GBM cells [72]. For example, adding a PD1 monoclonal antibody to a dendritic cell vaccine therapy increased T cell activation and produced a better response than the DC vaccine alone in a murine model of GBM [72].

Recently, combinatorial therapy targeting both VEGF and immune checkpoints has been investigated. This study addresses the low level of T cell infiltration in the GBM microenvironment. Targeting VEGF in GBM allows more T cells to infiltrate the tumor [88] while the ICI sustains activation of those infiltrating T cells [89]. Specifically, as anti-VEGF therapy was shown to lead to, an increased expression of PD-L1 on GBM cells hence by blocking PD1, it would be possible to circumvent this immune evasion [88]. In a murine model of GBM, while anti-PD1 monotherapy did lead to an increase in OS (27 days), the combination of anti-VEGF and anti-PD1 therapy lead to a greater increase in OS (46.5) [89].

Combining ICIs with standard of care provides an opportunity for a first line anti-tumor immune response. Unfortunately, the standard dose of TMZ is known to cause lymphopenia in patients [90]. The T cells that are present under the standard dose of TMZ express exhaustion markers including PD1 [90], but the low number of T cells makes it impractical to use ICIs to treat patients. When given a lower dose of TMZ more frequently, known as a metronomic dose, extensive lymphopenia is not observed [90]. Interestingly, while both standard dose and metronomic doses of TMZ resulted in some survival benefit to mice, PD1 monotherapy showed an increase in survival. This survival increase is preserved when mice are treated with both a metronomic dose of TMZ and anti-PD1 therapy as opposed to anti-PD1 therapy combined with standard dose TMZ [90]. This is likely due to the differences in T cell exhaustion and lymphopenia between the TMZ dose regimes. This study implies that combining ICI treatments with standard of care therapy is possible but that the dose to administer maybe of crucial consideration.

In a murine study examining anti-CTLA4 monotherapy, anti-PD1 monotherapy and anti-CTLA4/PD1 combinatorial therapy, 90% of mice given the combinatorial therapy survived to 90 days compared to 40% and 60% given anti-CTLA4 and anti-PD1 monotherapy, respectively [91]. This study also tested the effects of adding 1-MT, a drug which targets indoleamine 2,3 dioxygenase 1 (IDO1), to combinatorial ICI therapy. IDO1 has been shown to be expressed in GBM but not the normal brain [65] and evidence suggests IDO1 plays a role in immune evasion of GBM tumors [92]. When administered alone, 20% of mice treated with 1-MT showed long-term survival; however, when co-administered with combinatorial ICI therapy, 100% of mice showed long-term survival [91]. Combinatorial ICI therapy with and without the addition of 1-MT in larger, more established tumors was able to produce a 78% long-term survival rate in mice [91]. This is in line with the notion that multiple immune checkpoints may need to be targeted in GBM at a time.

As discussed previously, macrophages contribute to the immunosuppressive microenvironment of GBM tumors. As such, therapeutic strategies that combine ICIs with the inhibition of myeloid cells may prove effective in GBM. For example, AXL is a receptor tyrosine kinase found to be highly expressed in some GBM tumors; protein S, a ligand for AXL, is secreted by GAMs and promotes AXL signaling especially upon use of nivolumab [92]. Combining nivolumab with a small molecule inhibitor of AXL prolonged survival in an immunocompetent murine GBM model [92]. In another murine study, the combination of dendritic cell vaccine, anti-PD1 monoclonal antibody and a CSF-1R inhibitor significantly increased the survival of the mice [72]. Administration of the DC vaccine increased lymphocyte infiltration; GAMs responded by increasing PD-L1 expression. Treatment with an anti-PD1 monoclonal antibody did not increase activation of infiltrating lymphocytes. Adding a CSF-1R inhibitor lead to a decrease in infiltrating myeloid cells and an increase in lymphocyte infiltration. Collectively, using the combinatorial treatment significantly increased the long-term survival in mice bearing GBM tumors [72].

ICIs have also been studied alone. In a murine study assessing the effect of blockade of CTLA4, PD1 and PD-L1 individually, PD1 blockade was most effective with a 56% long-term survival rate; when combined with CTLA4 blockade, the long-term survival rate increases to 75% [93]. Additionally, the study demonstrated immunological memory in mice that received ICIs [93].

Taken together, these studies provide strong preclinical evidence for the efficacy of ICIs alone or in combination in treating GBM. Unfortunately, the mouse models have not been predictive of clinical efficacy yet.

## 5. Discussion

GBM has not responded well to ICI treatment alone but ICIs have shown more promise in an adjuvant therapy setting [14,15,16]. We have discussed the principal barriers to GBM treatment that all likely affect clinical responses in GBM. An additional barrier, which needs investigation in the context of immunotherapies, is the diffuse nature of GBM tumors. GBM is characterized by diffuse infiltration into the surrounding brain [1]. This infiltration cannot be visualized by MRI. Evidence suggests that GBM-associated macrophages aid in the infiltration of GBM cells throughout the brain [94]. It is not currently known if immunotherapeutics, including ICIs, have any effect on these infiltrating cells or indeed whether GBM-targeted immune cells will be able to move through the brain to reach these cells. However, a murine study utilizing dentritic cell vaccination with and without PD1 inhibition as a treatment for GBM showed that PET scans can be used to non-invasively monitor immune responses within the tumor itself [95]. This imaging technique utilizes imaging substrates produced by activated immune cells and was able to illuminate TILs [95]. This imaging technique may enable detecting treatment response in infiltrating tumor cells.

Considering the BBB/BBTB barriers, intratumoral or local delivery of drugs, specifically immunotherapeutics, has been previously suggested by us and others [55,61]. There are currently two clinical trials which deliver therapeutics via direct injection into the resection cavity, both of which involve IV delivery of an ICI as a combinatorial strategy [81,83]. Bypassing the BBB/BBTB through the use of CED allows for a more controlled administration of therapeutic compounds and can prevent some of the systemic side effects that many drugs cause. It remains to be seen whether loco-regional delivery empowers the ICIs to exert their desired effects.

Many of the immunotherapeutics discussed in this review are targeting the T cell anti-tumor immune response. However, it is important to explore other aspects of immune suppression in order to fully harness the patient’s immune system to fight off the tumor. A part of this immunosuppression is exerted by the immune cell infiltrates like myeloid cells or macrophages. For example, there is an abundance of evidence that the macrophages present in the GBM tumor microenvironment contribute significantly to the immune evasion of the tumor and silencing of T cells [59,60,63,67,69]. Further understanding of the interactions between GBM cells, TAMs, and T cells may provide new targets for novel immunotherapeutic strategies.

In addition, monotherapy as well as combinatorial therapy has shown efficacy in preclinical studies [65,72,88,89,90,91,93]. Treatment strategies involving ICIs in combination with other therapies have started to be tested in the clinic [73,75,76,79,96]. Many of these new strategies combine an ICI with a treatment or method, which incites an immune response. This would allow for activated T cells to be recruited to the tumor while administering an ICI would prevent the suppression of these T cells and would extend the immune response prompted by the initial signal. Our lab and others seek to improve upon this strategy.

Current research examines whether ICIs can become an important component in a combinatorial immunotherapy of GBM. The clinical trials that are active address at least one of the barriers to treatment discussed in this review. They combine immune stimulatory treatments with ICIs as well as deliver treatments loco-regionally in order to bypass the BBB. There are at least two trials which involve assessment of treatment-related biomarkers which will provide valuable insight into the response of GBM to combinatorial immunotherapy. However, there seems to be a long way to go before we will see a significant effect of ICIs in GBM patients.

## Figures and Tables

**Table 1:** FDA approved immune checkpoint inhibitors.

Drug	Immune Checkpoint Targeted	Year of Initial FDA Approval	Approved For:
Ipilimumab (Yervoy)	CTLA4	2011	Unresectable or metastatic melanoma; cutaneous melanoma; renal cell carcinoma (in combination with Nivolumab)
Nivolumab (Opdivo)	PD1	2014	Melanoma; Metastatic non-small cell lung cancer; Metastatic small cell lung Cancer; Advanced renal cell carcinoma; Hodgkin lymphoma; head and neck squamous cell carcinoma; advanced/metastatic urothelial cancer; colorectal cancer; hepatocellular carcinoma;
Pembrolizumab (Keytruda)	PD1	2014	Melanoma; non-small cell lung cancer; small cell lung cancer; head and neck squamous cell carcinoma; Hodgkin lymphoma; primary mediastinal large B-cell lymphoma; urothelial carcinoma; gastric cancer; esophageal cancer; cervical cancer; hepatocellular carcinoma; Merkel cell carcinoma; renal cell carcinoma; endometrial carcinoma
Atezolizumab (Tecentriq)	PD-L1	2016	Urothelial carcinoma; non-small cell lung cancer; Triple negative breast cancer; Small cell lung cancer
Avelumab (Bavencio)	PD-L1	2017	Metastatic Merkel cell carcinoma; urothelial carcinoma; advanced renal cell carcinoma (in combination with axitinib)
Durvalumab (Imfinzi)	PD-L1	2017	Advanced/metastatic urothelial carcinoma; unresectable stage III non-small cell lung cancer

**Table 2:** Clinical trials investigating the use of ICIs for treatment of GBM.

Name	NCT ID	Phase	Planned Enrollment	Arms
Neoantigen-based Personalized Vaccine Combined with Immune Checkpoint Blockade Therapy in Patients with Newly Diagnosed, Unmethylated Glioblastoma	NCT03422094	I	30	Cohort A: NeoVax + Nivolumab (at progression) Cohort B: NeoVax + Nivolumab (at Cycle 2) Cohort C: NeoVax + Nivolumab (at Cycle 1)
Avelumab in Patients with Newly Diagnosed Glioblastoma Multiforme	NCT03047473	II	30	Addition of Avelumab to standard treatment
Combination Adenovirus + Pembrolizumab to Trigger Immune Virus Effects (CAPTIVE)	NCT02798406	II	49	Intratumoral DNX-2401 followed by IV Pembrolizumab
GMCI, Nivolumab, and Radiation Therapy in Treating Patients with Newly Diagnosed High-Grade Gliomas	NCT03576612	I	36	Cohort 1: MGMT Unmethylated patients; AdV-tk injection into resection cavity, valacyclovir 14 days, radiation after 8 days, TMZ after valacyclovir, Nivolumab every 2 weeks to 52 weeks Cohort 2: MGMT Methylated and undetermined patients; AdV-tk injection into resection cavity, valacyclovir 14 days, radiation day 8, TMZ after valacyclovir, Nivolumab every 2 weeks to 52 weeks.
Laser Interstitial Thermotherapy Combined with Checkpoint Inhibitor for Recurrent GBM	NCT03277638	I/II	34	Arm 1: IV Pembrolizumab 7 days pre-surgery with LITT Arm 2: IV Pembrolizumab 14 days post-surgery with LITT Arm 3: IV Pembrolizumab 35 days post-surgery with LITT
Translational Study of Nivolumab in Combination with Bevacizumab for Recurrent Glioblastoma	NCT03890952	II	40	Arm A: Nivolumab + Bevacizumab in patients not undergoing salvage surgery Arm B: Nivolumab + Bevacizumab in patients undergoing salvage surgery

## Abbreviations

GBM	Glioblastoma
BBB	Blood-Brain Barrier
BBTB	Blood-Brain Tumor Barrier
ICI	Immune Checkpoint Inhibitor
IDO1	Indoleamine 2,3 Dioxygenase 1
PD1	Programmed Death 1
CTLA4	Cytotoxic T Lymphocyte Associated protein 4
PD-L1/L2	Programmed Death Ligand 1/Ligand 2
